# Supplementary material for: Microbiome and fragmentation pattern of blood cell-free DNA and fecal metagenome enhance colorectal cancer micro-dysbiosis and diagnosis analysis: a proof-of-concept study
Source: mSystems. 2025 Apr 29;10(5):e00276-25. doi: 10.1128/msystems.00276-25 (PMC12090784; doi:10.1128/msystems.00276-25)

**Supplementary figure legends**

**Figure S1.** Community composition of the blood microbiome. Community composition at the phylum level (A); community composition at the species level (B); the dominant phyla in three groups (C); the top ten differential KEGG pathways (Wilcoxon test, ***, *P* < 0.001) (D).

**Figure S2.** Correlation analysis between blood and fecal microbiome. Heatmap of Pearson correlation coefficients (A) and related P values (B) of the 177 shared species.

**Fig. S1**


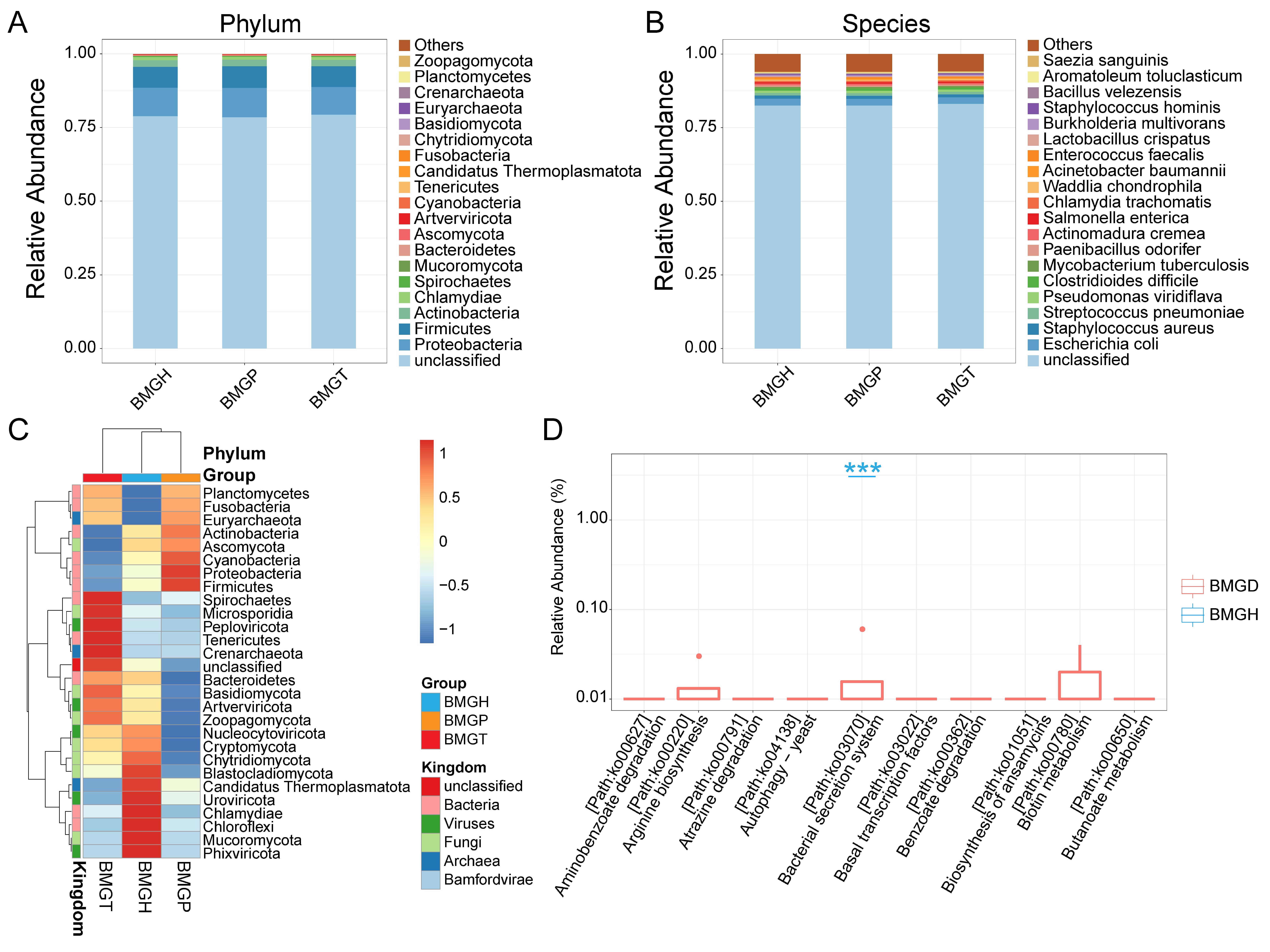


**Fig. S2**


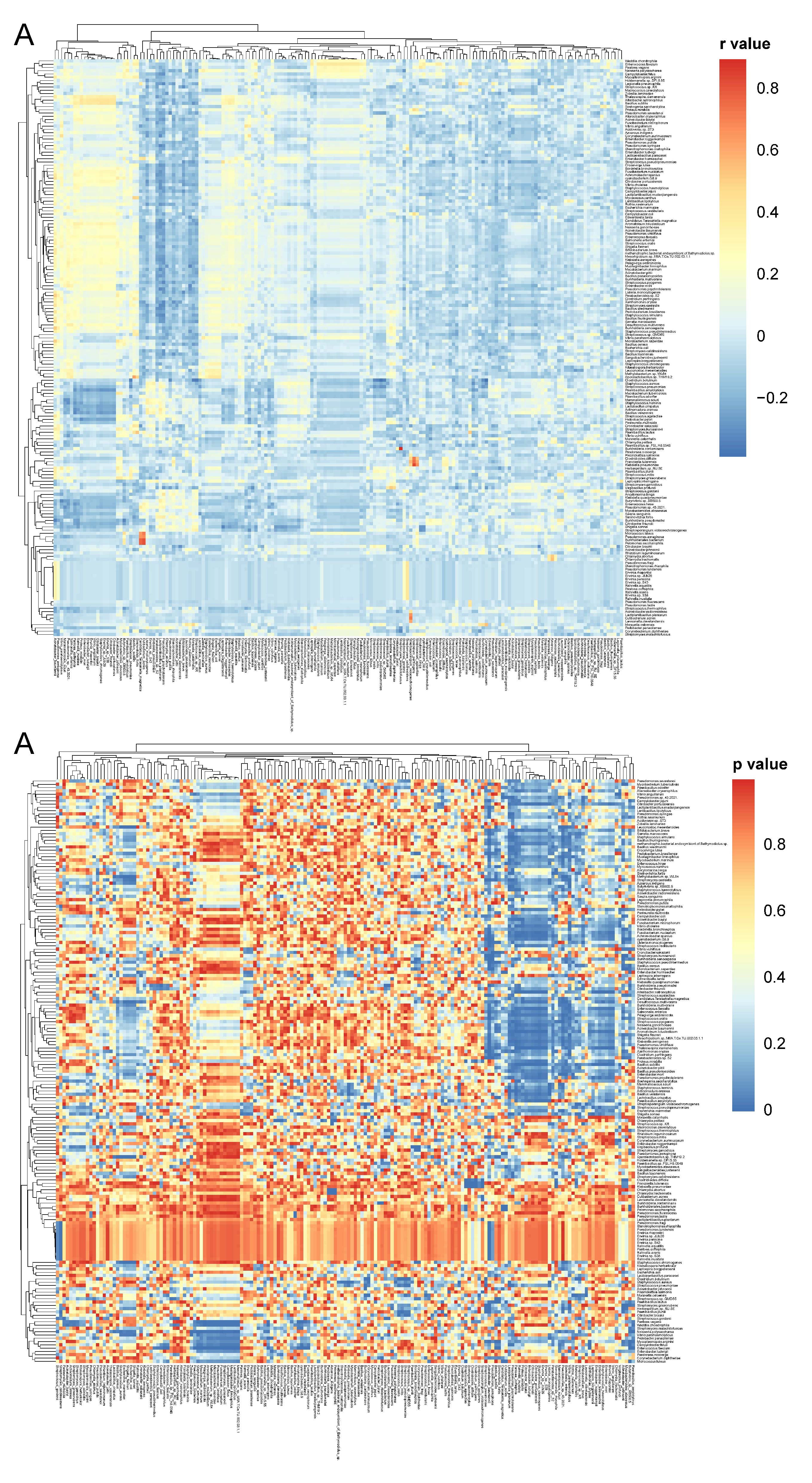

Supplement: Supplemental material — Figures S1 and S2. [file msystems.00276-25-s0001.docx]
